# Differential regulation of skeletal stem/progenitor cells in distinct skeletal compartments

**DOI:** 10.3389/fphys.2023.1137063

**Published:** 2023-02-28

**Authors:** Jea Giezl Niedo Solidum, Youngjae Jeong, Francisco Heralde, Dongsu Park

**Affiliations:** ^1^ Department of Biochemistry and Molecular Biology, College of Medicine, University of the Philippines Manila, Manila, Philippines; ^2^ Department of Molecular and Human Genetics, Houston, TX, United States; ^3^ Center for Skeletal Biology, Baylor College of Medicine, Houston, TX, United States

**Keywords:** periosteum, bone marrow, growth plate, bone regeneration, skeletal stem/progenitor cells, sutures, skeletal compartments, homeostasis

## Abstract

Skeletal stem/progenitor cells (SSPCs), characterized by self-renewal and multipotency, are essential for skeletal development, bone remodeling, and bone repair. These cells have traditionally been known to reside within the bone marrow, but recent studies have identified the presence of distinct SSPC populations in other skeletal compartments such as the growth plate, periosteum, and calvarial sutures. Differences in the cellular and matrix environment of distinct SSPC populations are believed to regulate their stemness and to direct their roles at different stages of development, homeostasis, and regeneration; differences in embryonic origin and adjacent tissue structures also affect SSPC regulation. As these SSPC niches are dynamic and highly specialized, changes under stress conditions and with aging can alter the cellular composition and molecular mechanisms in place, contributing to the dysregulation of local SSPCs and their activity in bone regeneration. Therefore, a better understanding of the different regulatory mechanisms for the distinct SSPCs in each skeletal compartment, and in different conditions, could provide answers to the existing knowledge gap and the impetus for realizing their potential in this biological and medical space. Here, we summarize the current scientific advances made in the study of the differential regulation pathways for distinct SSPCs in different bone compartments. We also discuss the physical, biological, and molecular factors that affect each skeletal compartment niche. Lastly, we look into how aging influences the regenerative capacity of SSPCs. Understanding these regulatory differences can open new avenues for the discovery of novel treatment approaches for calvarial or long bone repair.

## 1 Introduction

The skeletal system is among the largest of the human organ systems, constituting up to 15% of the total human body weight (Su et al., 2019). It allows functional body movement, protects internal organs, and serves as reservoir for minerals (Mizokami et al., 2017; Su et al., 2019); bones also have extra-skeletal endocrine functions (Su et al., 2019) that are essential for overall body homeostasis and systemic health (Ambrosi et al., 2019). Skeletal system functions are affected by skeletal shape, strength, and stiffness, which substantially change with the stage of development and age (Sheehan et al., 2018).

Advancing age is a key risk factor for degenerative bone and cartilage disorders, such as osteoporosis and osteoarthritis (Raisz and Seeman, 2001; Su et al., 2019; Jeong and Park, 2020), which lead to decreased mobility and diminished quality of life (Lee et al., 2014). However, bone mass, strength, and vitality are affected by other factors aside from age (Demontiero et al., 2012; Nandiraju and Ahmed, 2019). Alterations in cellular components, hormonal, biochemical, and vasculature status, which can be brought about by metabolic disorders, are examples of intrinsic factors (Demontiero et al., 2012), whereas nutrition, physical activity, injury, and comorbidities are some of the contributing extrinsic factors (Demontiero et al., 2012; Sheehan et al., 2018). Congenital or acquired skeletal deformities also affect the form and function of the skeletal system due to geometric abnormalities of the bones and articulating surfaces (Sheehan et al., 2018).

Some age-related defects in bones and cartilage have been attributed to changes in the populations and functions of stem cells in skeletal tissues (Jeong and Park, 2020). These molecular and functional changes in skeletal stem/progenitor cells (SSPCs) lead to a negative bone balance with reduced bone remodeling coupled with continued, or even accelerated, bone resorption (Demontiero et al., 2012). By itself, stem cell regeneration of large skeletal defects is difficult and often lead to the delay or failure of bone repair (Vidal et al., 2020). Confounded by aging and age-related diseases (e.g., diabetes), incidence of bone fractures and failure of large bone defect repair is further amplified. (Clark et al., 2017; Wu et al., 2021).

Currently, the goals of therapies for degenerative bone conditions are fracture prevention and decreased bone resorption through antiresorptive agents. For the reconstruction of critical-sized bone defects, transplantation of an autologous free vascularized bone flap containing the patient’s cells, growth factors, and a vascularization bed is the current gold standard approach (Vidal et al., 2020). However, these vascularized bone flaps are of finite supply, and their harvest can result in significant morbidity and anatomical incompatibility. Prosthetic and bio-matrix materials are also unable to restore complex sensory and motor functions, do not expand with age, and present a risk of failure and infection (Borrelli et al., 2020; Tang et al., 2021). Recently, in the field of tissue engineering and regenerative medicine, the use of SSPCs in combination with scaffolds and growth factors has been introduced to facilitate bone regeneration (Miller et al., 2017; Borrelli et al., 2020; Tang et al., 2021). Therefore, a better understanding of the properties and regulation of SSPCs with respect to their locations and skeletal compartments, as well as the effects of age, can potentially facilitate the discovery of new approaches to bone defect reconstruction and degenerative bone disease therapy.

**FIGURE 1:**
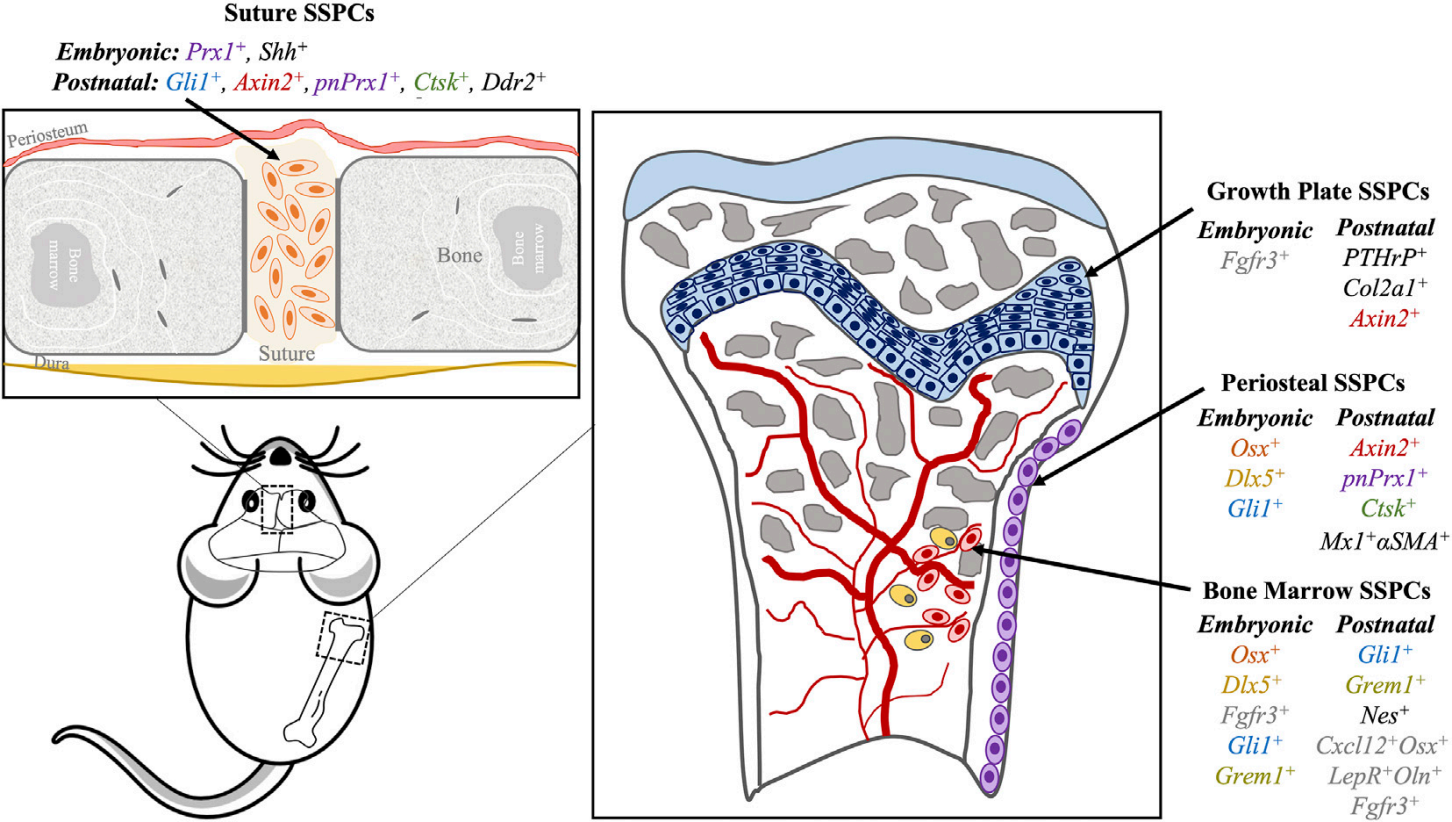
Skeletal stem progenitor cells (SPPCs) in different bone compartments in mice. Several markers of SSPCs per location (suture, growth plate, periosteum, bone marrow) are summarized here. Similar markers have similar text colors (i.e., *Axin2, Ctsk, pnPrx1 and Gli1*).

SSPCs are essential for skeletal development, bone remodeling, and bone repair and are characterized by the capacity for self-renewal and multipotency (Matsushita et al., 2020c). Traditionally, they have been known to reside within the bone marrow (BM), but recent scientific advancements identified distinct SSPC populations in various skeletal compartments such as the growth plate (GP), periosteum, and calvarial sutures (Matsushita et al., 2020c; Jeong and Park, 2020) (Figure 1). Adult SSPCs are heterogeneous, and each population potentially contributes to bone maintenance and regeneration in a different manner (Ortinau and Park, 2021). The cellular and matrix environment of each distinct SSPC population is also believed to regulate SSPC stemness and to direct its roles at different stages of development, homeostasis, and regeneration (Matsushita et al., 2020c). However, it is largely unknown how these SSPCs are regulated, and which specific roles they play in these biological processes (Iaquinta et al., 2019; Ortinau and Park, 2021). In this review, we present the different regulation mechanisms during the development and repair of the distinct SSPC populations in major compartments, namely, the suture, GP, periosteum, and BM. We also discuss the currently known changes that occur in the regulation pathways of SSPCs with aging.

## 2 Embryogenic cellular origins and location of SSPCs

**FIGURE 2:**
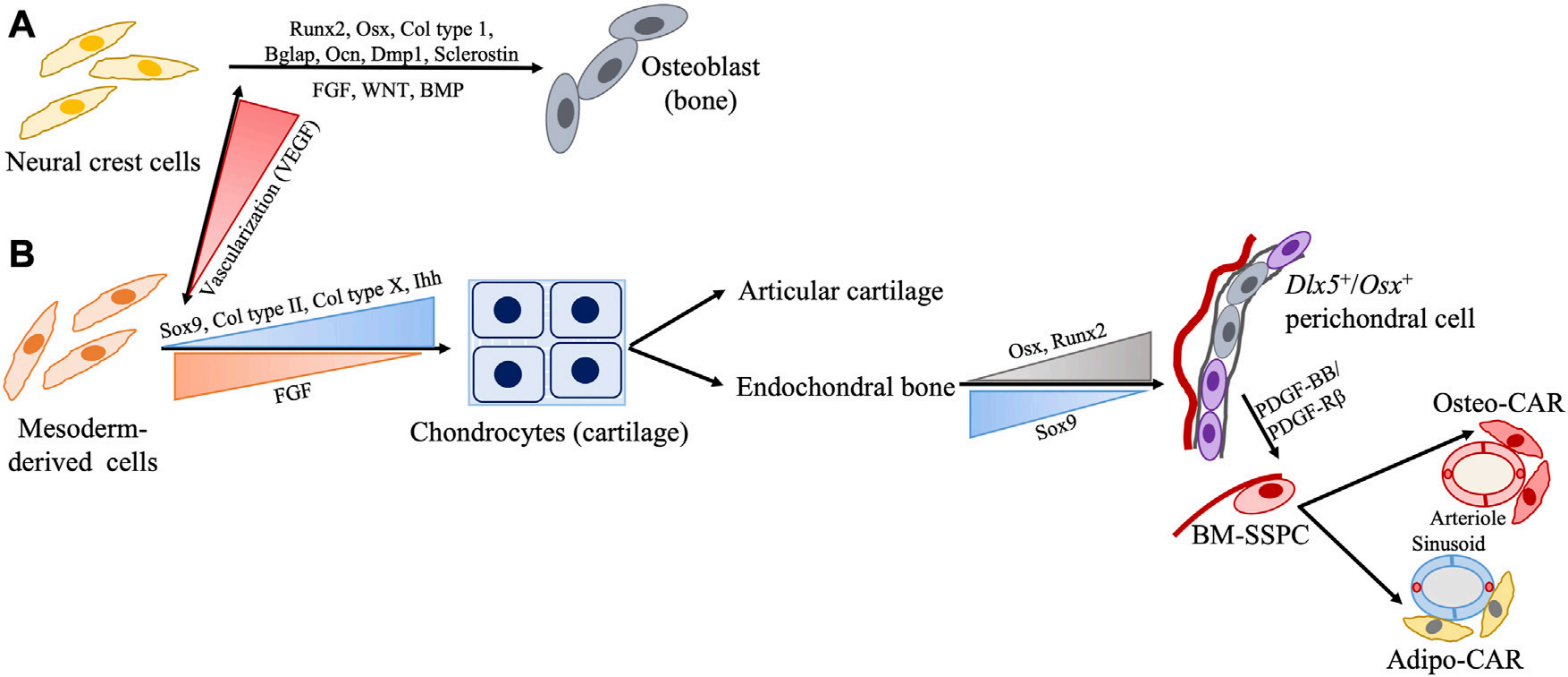
Schematic representation of SSPC regulation during development from embryonic cell origin **(A)** neural crest cells (NCC) and **(B)** mesoderm-derived cells (MDD). Generally, bones NCC form *via* intramembranous ossification while bones from the MDD form *via* endochondral ossification with few interesting exceptions such as NCC-derived clavicle forming *via* endochondral and MDD-derived parietal bone forming *via* intramembranous processes. Presence or absence of adequate vascularization may play a role in this.

**FIGURE 3:**
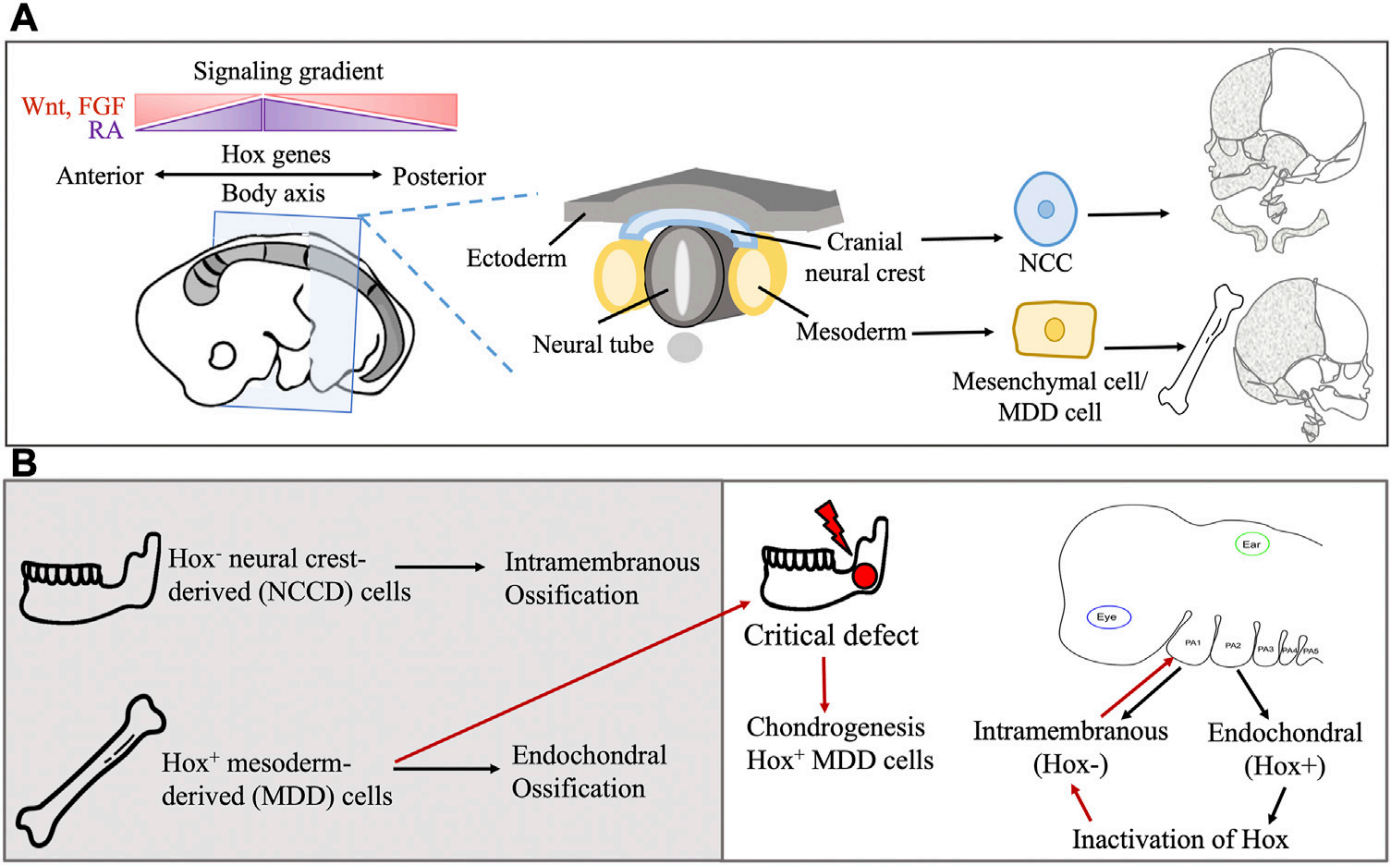
**(A)** Embryonic origin of extracranial and craniofacial bone and the role of Hox genes in directional differentiation. Hox genes also interact with regulatory gene, transcription factors and signaling molecule gradient for patterning of the body axis. **(B)** The Hox gene confers spatial regulation and affects the type of bone ossification during development and transplantation in bone defect.

The musculoskeletal system develops from various embryonic origins, including: 1) the paraxial mesoderm, 2) the parietal layer of the lateral plate mesoderm, and 3) the neural crest cells (NCCs), which undergo mesenchymal condensation to begin bone formation (Figures 2, 3) (Mitchell and Sharma, 2009). Most facial bones, the cranial vault, clavicle, and calvarial frontal bones originate from NCCs through the intramembranous ossification process. By contrast, most of the remaining bones in the skull and all perpendicular bones develop from mesoderm-derived (MDD) cells through the endochondral ossification process (Chung et al., 2009; Sadler and Langman, 2012; Schoenwolf et al., 2015; Moore et al., 2016). Some intriguing tissues include the clavicle originating from NCCs but forms through mixed intramembranous and endochondral ossification, and the calvarial parietal bones which originate from MDD but are formed through the intramembranous ossification process (Percival and Richtsmeier, 2013). Intramembranous bones develop *via* direct osteoblast differentiation within the mesenchyme, while endochondral bones develop with an intermediate cartilage structure before being replaced by or transformed into bones (Sadler and Langman, 2012; Schoenwolf et al., 2015; Moore et al., 2016; Galea et al., 2021; Shu et al., 2021). Furthermore, mesodermal cells from different embryonic origins show different transcriptomic signatures and differentiation potentials, suggesting that tissue-specific SSPCs with different embryonic origins are present in different bones and that they require differential regulation pathways for bone regeneration (Sacchetti et al., 2016).

Intramembranous bone formation begins with the expression of *Runx2* driving SSPCs into the osteoblast lineage (Pazhanisamy, 2013) (Figure 2). This is followed by the sequential expression of *osterix* (*Osx*), *type I collagen*, *Bglap* (or *osteocalcin*), and *Spp1* (or *osteopontin*), which are the core osteoblast differentiation factors. As osteoblasts become surrounded by bone matrix, they express late markers such as the *dentin matrix protein 1* (*Dmp1*). Lastly, the expression of the osteocyte marker *sclerostin* is observed (Pazhanisamy, 2013; Galea et al., 2021). In endochondral bone formation, however, *Sox9* initiates chondrocyte commitment in the pre-condensing mesenchyme. During early chondrocyte development, *Sox9, Sox5*, and *Sox6* are highly expressed and subsequently drive the expression of early cartilaginous matrix components *type II collagen* (*Col II*) and *aggrecan* (Galea et al., 2021). During the terminal hypertrophic stage of endochondral ossification, co-expression of cartilaginous (*type X collagen*) and osteoblastic (*Runx2*, *Osx*, *bone sialoprotein*) markers is observed. *Sox9* expression persists in early hypertrophic chondrocytes and induces the expression of *type X collagen* while inhibiting *Runx2* activity. Later on, the degradation of *Sox9* protein relieves the inhibition of *Runx2*, thus allowing for chondrocyte-osteoblast transformation and subsequent mineralization (Taher et al., 2011; Galea et al., 2021). Overall, endochondral and intramembranous ossification use distinct molecular signals responsible for the different types of bone formation.

The expression of regulatory genes in specific cell types and locations in the body may also account for the differences in SSPC functionality. For example, a mandibular injury site undergoes osteogenic regeneration through *Homeobox* non-expressing (*Hox* ^*−*^) neural crest-derived (NCD) cells, while tibial or long bone injuries ossify with *Hox^+^* MDD cells (Lappin et al., 2006) (Figure 3). *Hox* genes encode the Hox proteins, which are master regulators of embryonic development, but these genes continue to be expressed throughout postnatal life. In humans, they control body proportions, vertebral segments, and proper spatial development of organs and limbs (Lappin et al., 2006; Rux and Wellik, 2017). Upon transplantation of MDD cells to a mandibular injury site, *Hox^+^* MDD cells remain *Hox^+^*, while inherently *Hox* ^*−*^ NCD cells transplanted to the tibial injury site become *Hox^+^*. This indicates a sense of positional identity that is unchanged with transplantation, and this positional identity is also seen in facial bone development (Leucht et al., 2008). The first pharyngeal arch (PA) cells, which are *Hox* ^*−*^ *,* form most of the facial bones in an intramembranous manner. The second PA cells, which are *Hox^+^,* form the hyoid, styloid, and stapes bones in an endochondral manner (Weber et al., 2021). Inactivation of *Hox* genes in the second PA results in intramembranous ossification, while their overexpression in the first PA results in second PA-like elements (Bonaventure, 2001; Kitazawa et al., 2022). Overall, these findings suggest that embryonic cell origin may confer a differentiation bias to SSPCs.

Although some distinct SSPCs may come from the same embryonic cells, they undergo further development in their distinct skeletal compartments. With the complex development in each compartment, dynamic and specialized microenvironments are also formed (Kurenkova et al., 2020). Most likely, these microenvironments provide an additional layer of unique regulation to each SSPC population aside from what is offered by embryonic cell origin (Donsante et al., 2021) (Table 1). Parallel niches may therefore be progressively altered, explaining the different properties and functions of distinct SSPCs through time and condition, which will be discussed later.

**TABLE 1:** Summary of the regulations of distinct skeletal stem//progenitor cells (SSPCs) in different skeletal compartments during development, remodeling and aging.

Skeletal Stem//Progenitor cells	Molecular regulators	Development	Remodeling	Aging
Suture	EphA4	-Directs embryonic osteoprogenitor cell migration Ting et al. (2009); Ishii et al. (2015)	?	-Closes between the second and third decade of life Libby et al. (2017)
	Twist1, FGF, Notch	-Maintains cell stemness Bonaventure (2001); Ishii et al. (2015)	?	
	BMP2/4/7, FGF-2, FGFR-1, IGF-2, Ptn, Sparc, Oc (from dura mater)	-Promotes interstitial bone formation during brain and skull growth Wan D C et al. (2008)	-Potentially the same	
	TGFβ	-Maintains a continuous osteogenic lineage commitment Ishii et al. (2015)	-Promotes osteogenic differentiation Wilk et al. (2017)	
		- Triggers interstitial bone production Wang et al. (2020)		
*Gli1^+^*	Hh, BMP/Bmpr1a	-Promotes osteogenic differentiation adding osteoblasts in the osteogenic font for interstitial growth Zhao et al. (2015)	-Promotes osteogenic differentiation Zhao et al. (2015)	-
	Hh/RANKL	?	-Stimulates osteoclast differentiation and resorption activity Guo et al. (2018)	
*Axin2* ^+^ Maruyama et al. (2010); Maruyama et al. (2017); Maruyama et al. (2021)	BMP/Bmpr1a/Rapb1	-Suppresses osteogenesis and maintains cell stemness	?	-
	FGF/FGFR1	-Enhances osteoblast proliferation and differentiation	?	
	Wnt/β-catenin	-Mediates BMP/FGF balance	-Promotes cell fate switch between osteoblast and chondrogenic lineage cells during injury, thus may promote osteochondral regeneration.	
	Wnt3	?	-Increased calvarial bone regeneration	
*pnPrx1* ^+^ Wilk et al. (2017)	Wnt	-Inhibited by *Dkk1* and *Sost*	-Promotes osteogenic differentiation	-
*Ctsk* ^+^	Bmp 1/2, Runx2, Sox9 Debnath et al. (2018)	?	-Potentially promotes osteogenic differentiation	?
*Ddr2* ^+^ Greenblatt et al. (2021)	Myc, Runx2, Klf4, Nes1, Msx1/2, Acta2, Lgr5, and Lrig1	-Potentially maintains stem cell quiescence and suture patency	?	?
	Sox9, Col2a1, and Acan	?	-Potentially promotes endochondral ossification	
Growth Plate				-Closes near puberty Setiawati and Rahardjo (2019)
*Fgfr3*+ (embryonic)	IHh	-Promotes chondrocyte proliferation	-	-
		-Promotes chondrocyte hypertrophy Mizuhashi et al. (2018); Ağırdil (2020)		
*PTHrP+* (postnatal)	PTHrP	-Suppresses hypertrophic differentiation of proliferating chondrocytes Mizuhashi et al. (2018)	-Same as development	-
	PTHrP, Runx2, BMP, IHh, TGFβ	-Promotes chondrocyte proliferation		
		-Promotes chondrocyte hypertrophy Mizuhashi et al. (2018); Ağırdil (2020)		
*Col2a1+* (postnatal)	Notch Zieba et al. (2020)	-Maintains SSPC population and functions	-Notch2 allows hypertrophy and mineralization of proliferating chondrocytes.	
		-Notch 1 Promotes chondrocyte proliferation, GP organization and hypertrophy		
	mTORC1, IGF-1	-Increased number and thickness of multi-columnar clones Newton et al. (2019)	?	-
	Gsα, Gq/G11α	-Maintains quiescence of resting chondrocytes Guo et al. (2009); Chagin et al. (2014)		
*Axin2* ^+^ (postnatal)	Wnt/β-catenin	-Physiologically inhibited in the resting zone. Maintenance of SSPCs in the resting zone	-Same as development	-
		-Promotes chondrocyte proliferation and hypertrophy in the proliferating zone Hallett et al. (2021)		
Periosteum	OSTN/CNP/GC-B signaling (towards growth plate)	-	-Chondrocyte proliferation and maturation Watanabe-Takano et al. (20199	?
	IHh	-Regulates chondrocyte proliferation and differentiation Wang et al. (2020)	-Same as development	?
	PGE2, Postn (from mechanical loading)	-	-Higher mineralization and apposition rate Galea et al. (2011); Bivi et al. (2013); Gerbaix et al. (2015)	-Decreased loading causes bone resorption and osteocyte apoptosis Lloyd et al. (2012)
*Dlx5+*, *Osx+, Gli1+* (embryonic)	HIF-1α, VEGF	-Absence may promote expansion in periosteum and inhibition of migration to BM Nagao et al. (2017)	-	-
*Ctsk* ^+^ (postnatal)	Bmp, Runx2, Sox9, Wnt Debnath et al. (2018)	?	?	?
	LKB1	- May promote quiescence Han et al. (2019)	?	
	mTORC1	- Promotes appositional growth Han et al. (2019)	-Activated, couple with VEGF. Potential mechanism for osteochondral regeneration Wan C et al. (2008); Wan et al. (2010)	
*Axin2^+^* (postnatal)	Wnt	? (Possibly similar with *Axin2* ^+^ Su-SSPC) Maruyama et al. (2016); Ransom et al. (2016)	?	?
*Mx1* ^+^ *αSMA* ^+^(postnatal)	CCR5/CCL5	?	-Facilitates immediate migration to injury site Ortinau et al. (2019)	?
*pnPrx1* ^+^	TGFβ	-Inhibits adipogenesis Du et al. (2013)	?	?
	Prx1	-Inhibits the expression of *Osx* and *Runx2, and inhibits* osteogenic differentiation Lu et al. (2011)	-Reserved stem cells Duchamp de Lageneste et al. (2018); Esposito et al. (2020)	
	BMP/Cxcl12	?	-Activates injury induced SSPCs Esposito et al. (2020)	
	Postn, Sostdc1	-Maintains SSPCs pool Bonnet et al. (2013); Collette et al. (2013)	-Maintains SSPCs pool used for regeneration Bonnet et al. (2013); Collette et al. (2013)	
			-Inhibition hastens the expansion and differentiation of SSPCs Collette et al. (2013)	
	Notch/Jagged1 signaling	?	-Periosteal expansion of cortical bone young Youngstrom et al. (2016)	
Bone Marrow	Notch Vanderbeck and Maillard (2019); Sottoriva and Pajcini (2021)	-Maintains BM niche, promotes HSC maintenance, and promotes megakaryocyte/erythroid cell development	-Regulates hematopoietic recovery	?
	NO, IL-1, IL-6 (from M1 macrophage)	-May facilitate establishment and maintenance of BM niche Genin et al. (2015)	?	-Sustained exposure to inflammatory molecules Franceschi et al. (2018); Josephson et al. (2019)
	MAF/Runx2, Cbfβ, Forkhead box P1/CEBPβ	-Promotes osteogenesis, inhibits adipogenesis Wu et al. (2014); Wu et al. (2017); Li et al. (2017)	?	-Reduction of factors with aging releases inhibition to adipogenesis Li et al. (2017)
	MAF/PPARγ	?	?	-Promotes adipogenesis Li et al. (2017)
	RANKL/OPG	-	-Promotes osteoclastogenesis Weitzmann (2013); Zhang et al. (2020)	-Increased OPG production results in osteoclast differentiation Li et al. (2015)
	G-CSF (from B-lymphocytes)	-	-Promotes osteogenesis Weitzmann (2013); Zhang et al. (2020)	?
	IL-17 (from Th17 cells)	-	- Promotes osteogenesis in the long bones but suppression in calvarial bone Wang et al. (2020)	-Sustained exposure to inflammatory molecules Franceschi et al. (2018); Josephson et al. (2019)
	BMP2, TGFβ, osteopontin (from M2 macrophage)	-	-Promotes osteogenesis Chen et al. (2020)	-Sustained effects similar to remodeling
	IL-1α, TGFβ, ROS (from activated neutrophils)	-	-SSPCs differentiation into osteoblasts Nam et al. (2012); Lee (2013)	-Promotes negative bone balance or exhaustion of proliferating or differentiating cells Owusu-Ansah and Banerjee (2009); Chakkalakal et al. (2012)
*Dlx5* ^+^ (embryonic)	HIF-1α, VEGF	-Promotes angiogenesis needed for migration of BM-SSPCs from perichondrium to BM Nagao et al. (2017); Matsushita et al. (2022)	-	-
	IHh	-Bind to *Ptch1* promoting BM space formation Matsushita et al. (2022)	-	-
*Osx^+^(Gli1^+^)* (embryonic)	HIF-1α, VEGF	-Promotes angiogenesis needed for migration of BM-SSPCs from perichondrium to BM Nagao et al. (2017); Matsushita et al. (2022)	-	-
*Fgfr3* ^+^(*Grem1* ^+^) (embryonic)	IHh	-Promotes BM chondrocytes proliferation which may differentiate into osteoblasts for trabecular bone formation Matsushita et al. (2022)	-	-
*Gli1* ^+^ (postnatal)	Hh	-Promotes adipogenesis Shi et al. (2017)	?	?
	β-catenin	-Antagonizes pro-adipogenic Hh Shi et al. (2017)		
*Nes* ^+^(postnatal)	Cxcl12	-Maintains Wnt-inhibitory environment to maintain Seike et al. (2018)	?	?
		-Promotes quiescence of Cxcl12-abundant reticular (CAR) cells Méndez-Ferrer et al. (2010)		
*Grem1* ^+^ (postnatal)	Grem1/BMP	- Generates articular and growth plate cartilage Rowan et al. (2020)	?	?
		-Potentially promotes chondrogenesis Worthley et al. (2015)		
*Cxcl12^+^Osx^+^* (*Fgfr3* ^+^; *LepR* ^+^ *Oln* ^+^; osteogenic progenitor/osteo-CAR; postnatal)	Runx2	-Promotes osteoblast differentiation Shu et al. (2021)	-same as development	?
	Piezo1	-Promotes bone formation with mechanical loading Shen et al. (2021)	-same as development	-
*Cxcl12^+^Adiponectin^+^* (*Dlx5* ^+^; adipogenic progenitor/Adipo-CAR; postnatal)	Gs/cAMP/β-adrenergic signaling	?	-Potentially promotes BM adipocyte lipolysis, pre-adipocyte-like CAR cells differentiation, and osteogenesis Bachman et al. (2002); Lohse et al. (2003)	?
	Wnt/BMP/Bmpr1b signaling	?	- Potentially promotes pre-adipocyte-like CAR cells differentiation and osteogenesis Merrell and Stanger (2016); Matsushita et al. (2020a)	
	Cxcl12	?	-Attracts osteoblast and osteoclast progenitors into the BM Li et al. (2009); Yang et al. (2015)	
	Adiponectin	?	-Facilitates migration of osteoblast progenitors and repels osteoclast progenitors into injury site Li et al. (2009); Yang et al. (2015)	

*Gli1 - Zinc finger protein glioma-associated oncogene 1; Axin2 - Axis inhibition protein 2; pnPrx1 - Postnatal Paired-related homeobox protein; Ctsk - Cathepsin k; Ddr2 - Discoidin domain-containing receptor 2; Fgfr3 - Fibroblast growth factor 3; PTHrP - Parathyroid hormone-related protein; Col2a1 - Collagen type 2 alpha1; Dlx5 - Distal-less homeobox 5; Osx - Osterix; Mx1 - Myxovirus resistance 1; αSMA - α-Smooth muscle actin; Nes - Nestin; Grem1 - Gremlin 1; Cxcl12 - CXC motif chemokine ligand 12; LepR - Leptin receptor; Oln - Osteolectin*; EphA4 - Ephrin A receptor 4; FGF(R) - Fibroblast growth factor (receptor); BMP(r)- Bone morphogenic protein (receptor); IGF - Insulin-like growth factor; Ptn - Pleiotrophin; Sparc - Secreted protein acidic and cysteine rich; Oc/Ocn - Osteocalcin; TGFβ - Transforming growth factor-β; (I)Hh - (Indian) Hedgehog; RANKL – Receptor activator of NF-κB Ligand; Runx2 - Runt-related transcription factor 2; Sox9 - Sex-determining region Y-box transcription factor 9; Myc - Myelocytomatosis oncogene; Klf4 - Kruppel-like factor 4; Acta2 - Actin alpha 2; Lgr5 - Leucine-rich repeat-containing G-protein coupled receptor 5; Lrig1 - Leucine rich repeats and immunoglobulin like domains 1; Acan - Aggrecan; Gs - Guanine nucleotide-binding protein G subunit; Gq/G11α - G proteins Gqα and G11α; OSTN - Osteocrin; CNP - C-type natriuretic peptide; GC-B - Guanylate cyclase-B; PGE2 - Prostaglandin E2; Postn - Periostin; HIF-1α - hypoxia inducible factor-1α; VEGF - vascular endothelial growth factor; LKB1 - liver kinase b1; mTORC1 - mammalian target of rapamycin complex 1; CCL5 - CC motif chemokine ligand 5; CCR5 - CC motif chemokine receptor 5; Sostdc1 - Sclerostin domain-containing protein 1; NO - Nitric oxide; CEBPβ - CCAAT/enhancer-binding protein beta; Cbfβ - Core binding factor beta; MAF - Musculoaponeurotic fibrosarcoma; PPARγ - Peroxisome proliferator-activated receptor γ; cAMP - Cyclic adenosine monophosphate; OPG - Osteoprotegerin; G-CSF - Granulocyte colony-stimulating factor.; IL- Interleukin; ROS - Reactive oxygen species; Wnt - Wingless-related integration site.

## 3 Differences in the regulation of calvarial and long bone development and remodeling

Calvarial bone formation begins around the third week of gestation (Jha et al., 2018). At this stage, NCCs expand and form a condensed mesenchyme. Capillaries then begin to surround the mesenchymal condensation which may serve as a vehicle for nutrient supply, osteoblastic factor transport, and conduit of additional NCCs and SSPCs (Percival and Richtsmeier, 2013; van Gastel et al., 2020). Next, the cells in the mesenchyme center start to differentiate directly into osteoblastic cells and generate an osteoid (calvarial bone primordia) which later become mineralized bones (Ishii et al., 2015; Kenkre and Bassett, 2018). The Bone Morphogenic Protein (BMP 2/4/7) signaling pathway and its immediate-early effector homeodomain transcriptional factor (*Msx2*) play major roles in the early specification of the calvarial bone primordia from NCCs by positively controlling the expression of *Runx2*. Transcription factor *Foxc1*, on the other hand, negatively regulates *Msx2* and *Bmp2/4* and positively regulates *Noggin* to prevent premature differentiation of the frontal bone primordia, thus promoting apical migration of undifferentiated progenitor cells. Wnt signaling is a key regulator in the early specification of primordia that favors the osteogenic lineage. Wntless, a Wnt ligand transporter, found in the cranial surface ectoderm and underlying mesenchyme, promotes expression of the Wnt ligands (Wnt5a/11/4/3a/16) and secretion of Wnt protein, activating *Twist1* and then *β-catenin* downstream. *Β-catenin* promotes the osteogenic lineage but represses the chondrogenic lineage in the cranial mesenchyme. Interestingly, a haploid deficiency of *Fgfr1* in suture cells switches their fate to form ectopic chondrocytes in the suture mesenchyme, suggesting that local Fibroblast growth factor (FGF) signals are necessary for their direct intramembranous ossification (Maruyama et al., 2010).

Once the primordia are established, osteogenic precursors migrate, through the EphrinA (EphA) signaling, to the edge of the growing bone, where they contribute to the apical expansion of the calvarial rudiments. Wnt signaling is still a prerequisite at this point for the final phase of osteoblast differentiation; TGF-β signaling, on the other hand, is required to maintain a continuous osteogenic lineage commitment (Ishii et al., 2015). Between calvarial bones, cranial sutures develop while allowing calvarial expansion for brain growth (Sadler and Langman, 2012). A study by Deckelbaum et al. (2012) identified a group of Sonic hedgehog (Shh)-responsive cells in the head mesoderm as precursors of the coronal suture. These cells migrate first to the supraorbital ridge transiently expressing *En1*, a vertebrate homolog of the *Drosophila* transcription factor *engrailed*, before apically migrating together with the calvarial rudiments to form the coronal suture (Deckelbaum et al., 2012). Other embryonic origins of the suture precursor cells still need to be identified.

Long bone formation becomes visible by the end of the fourth week of gestation. Limbs initiate with small bud formation as outpocketing from the ventrolateral body wall. These limb buds generate a core of mesenchymal cells from the somatic layer of the lateral plate mesoderm covered by a layer of ectoderm. An apical ectodermal ridge (AER) is located at the distal end of the limb and induces rapid mesenchymal cell proliferation without differentiation. FGF signals in the so-called progress zone control proximal to distal limb growth (Bonaventure, 2001; Sadler and Langman, 2012; Schoenwolf et al., 2015; Moore et al., 2016). A unique feature of endochondral bone formation is the moment when the cells move further from the influence of the AER, causing local FGF levels to decrease and allowing the mesenchymal cells to differentiate into cartilage. This is where endochondral ossification begins, and skeletal compartments subsequently develop (Figure 2). GP formation starts off with early cartilage development through chondrocyte proliferation and differentiation of *Fgfr3* ^+^ cells in the mesenchymal condensation (Ono and Kronenberg, 2016; Zieba et al., 2020; Matsushita et al., 2022). The proliferating chondrocytes become mature and later organize, through Notch signaling, at both sides of long bones as a tri-layer GP consisting of resting, proliferating, and hypertrophic chondrocyte zones (Ono and Kronenberg, 2016; Zieba et al., 2020). The remaining cells form the outer layer called the perichondrium. All of the mesenchymal condensations in the forming limbs still remain avascular at this point (Percival and Richtsmeier, 2013). The spatio-temporal differences on angiogenesis may also explain the unique ossification processes with avascular state limiting supply of osteogenic factors and SSPCs that would promote osteogenesis (Percival and Richtsmeier, 2013; van Gastel et al., 2020). Eventually, hypoxic condition in limb forming cells promotes vascular invasion to the perichondrium leading to osteoblast differentiation, and development of perichondrium to periosteum and articular soft tissues (Percival and Richtsmeier, 2013). The periosteum becomes a layer of connective tissue housing the proliferating progenitor cells with chondrogenic and osteogenic differentiation properties, while the osteoblasts differentiating mostly from *Dlx5* ^+^ cells of the periosteum form a bony collar around the shaft of limb bones (Vanderbeck and Maillard, 2019; Sottoriva and Pajcini, 2021; Matsushita et al., 2022). Subsequently, the marrow cavity forms as long bones develop, and blood vessels invade the cartilage template from the osteogenic perichondrium, which are maintained through Notch signaling (Vanderbeck and Maillard, 2019; Sottoriva and Pajcini, 2021). Blood-borne hematopoietic progenitors and BM stromal cells then seed this environment. While most of the BM stromal cells originate from the outer perichondral *Dlx5* ^+^ cells, a minimal contribution of inner perichondrial *Osx* ^+^ cells and cartilage *Fgfr3* ^+^ cells implicate that BM stroma may have transitions from primitive progenitor cells in early postnatal development to definitive SSPCs in adult bone homeostasis, respectively. This is exemplified by the transition of fetal *Osx* ^+^ SSPCs to more long term postnatal *Osx* ^+^ BM SSPCs, and a shift from a more proliferative fetal *Dlx5* ^+^ SSPCs to a more quiescent postnatal *Dlx5* ^+^ BM-SSPCs. (Mizoguchi et al., 2014; Matsushita et al., 2022). With age, a subset of BM stoma cells further shifts towards adipocyte development (Taher et al., 2011; Bianco and Robey, 2015).

The difference between calvarial vs. long bone is apparently observed in bone injury healing. In general, all bones heal through three overlapping processes, namely, inflammation, bone formation, and bone remodeling (Sheen and Garla, 2021). Immediately after bone injury, a hematoma develops, leading to inflammation of the injury site. Inflammatory cells migrate into the injury site and secrete various cytokines and growth factors like tumor necrosis factor-α (TNF-α), BMPs, and interleukins, subsequently attracting more inflammatory and osteogenic progenitor cells (Wang et al., 2020; Sheen and Garla, 2021). Bone regeneration then begins with callus formation and a gradual decrease in inflammation. A unique process of long bone fracture healing is the fibrocartilaginous callus formation that first appears at days 5–11. During this process, vascular endothelial growth factor (VEGF) allows angiogenesis, and BMP drives the differentiation of SSPCs into chondroblasts, osteoblasts, and fibroblasts. At the same time, woven bones begin to appear adjacent to the periosteal layer (Wang et al., 2020; Sheen and Garla, 2021). Later on, on days 11–28, when Sox9 protein is degraded, inhibition of osteogenic *Runx2* is relieved, and a bony callus then forms as chondrocytes calcify with calcium–phosphate crystals, which is followed by bone replacement by osteoblasts (Sadler and Langman, 2012; Schoenwolf et al., 2015; Moore et al., 2016; Galea et al., 2021). In contrast, calvarial injury repair is normally completed by repeated cycle of bone resorption by osteoclasts and bone formation by osteoblasts without forming fibrocartilaginous callus (Lim et al., 2013; Wang et al., 2020), although some recent studies show endochondral ossification upon scaffold induced calvarial injury repair (Ko and Sumner, 2021). Macrophage-colony stimulating factor and receptor activator of nuclear factor kappa-B ligand (RANKL) are two critical cytokines for osteoclast differentiation (Castillo et al., 2017; Wang et al., 2020). These factors recruit osteoclast precursors, activate their fusion to form multinucleated pre-osteoclasts, and induce downstream signaling molecules (e.g., mitogen-activated protein kinase, TNF-receptor-associated factor 6, NF-κB, and c-fos) and key transcription factors (e.g., nuclear factor of activated T-cells [NFATc1]) that regulate osteoclast gene expression (Kenkre and Bassett, 2018).

In summary, calvarial and long bone development and remodeling leads to different bone morphologies and histologic characteristics. Although not all the regulatory pathways involved are the same (Table 1), Notch, BMP, TGFβ, Hedgehog (Hh) and Wnt/β-catenin signaling pathways play a key role in embryogenesis and regulation within the different bone compartments (Sottoriva and Pajcini, 2021). In addition, the balance between proliferation and differentiation is important in the development and remodeling of these compartments.

### 3.1 Unique regulation of calvarial suture SSPCs (Su-SSPCs)

**FIGURE 4:**
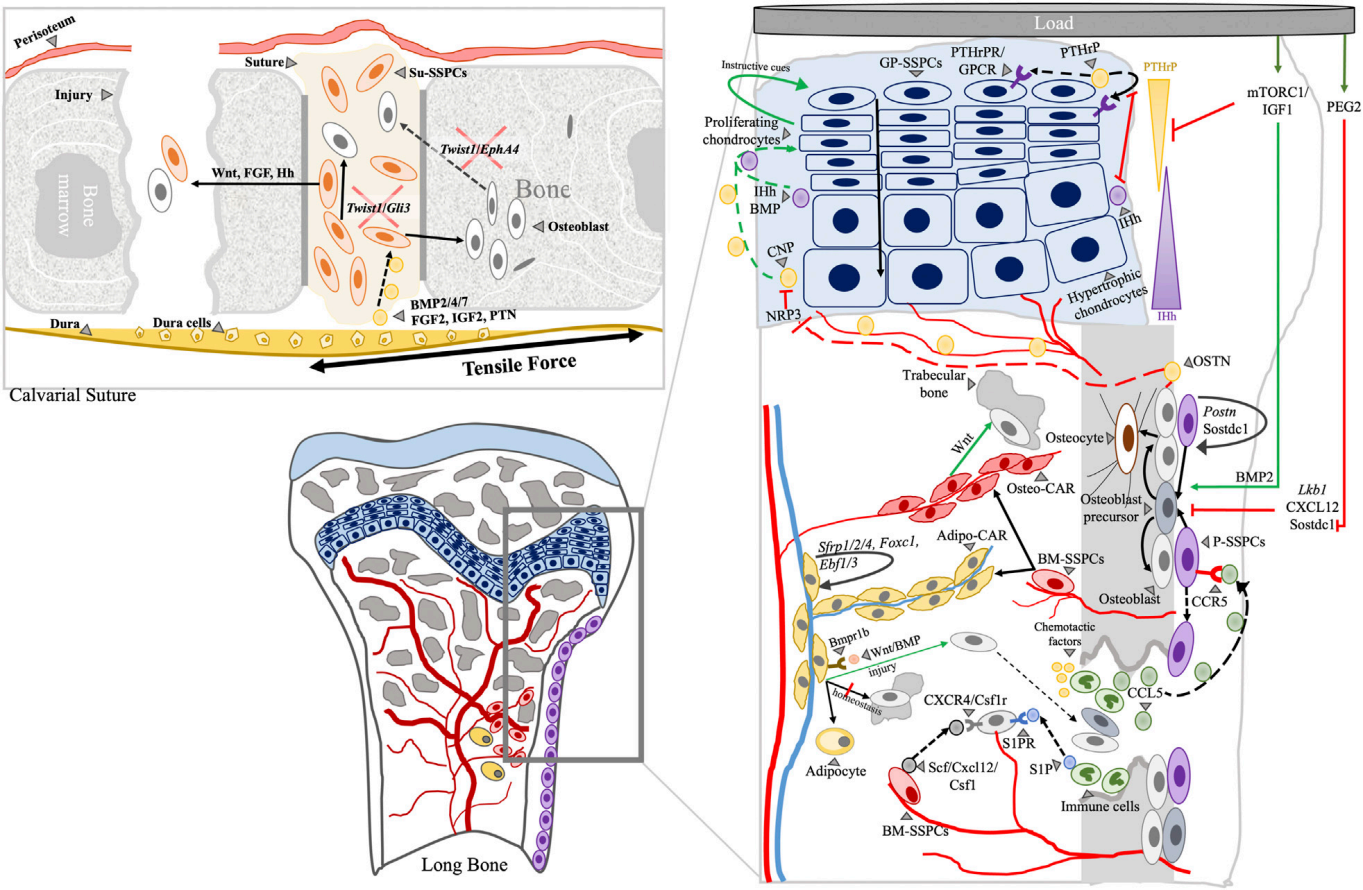
Schematic representation of plausible interactions of cellular components and molecular regulators at various skeletal compartments during bone remodeling and aging. Calvarial and long bone skeletal compartments are shown. Arrowhead: label; Black solid arrow: fate; Black dashed arrow: movement/migration; Red line: negative regulation; Green arrow: positive regulation; Arrows indicate directions of interactions or positive regulation.

Sutures of calvarial bones are unique structures that function as fibrous joints to facilitate calvarial bone movements and as brain cushions to absorb mechanical forces (Wang et al., 2020). With the growth of the brain, the meningeal and cutaneous periosteal layers grow in an ectocranial direction displacing the calvarial bones with them. The tensile physiological forces are then produced and serve as a stimuli to trigger interstitial bone production (Jin et al., 2016). During this process, skeletal stem/progenitor cells in sutures (Su-SSPCs) are a major contributor to calvarial bone growth in response to such forces (Wang et al., 2020) and express specific factors (e.g., *Runx2, Nel-like Molecule-1* [*NELL-1*], *TGFβ1*, and *FGF-2*) (Figure 4). Further, recent studies demonstrate that sutures act as the major sites of calvarial interstitial bone growth (Lana-Elola et al., 2007; Opperman et al., 2009; Jin et al., 2016) and constitute a unique microenvironment for adult craniofacial SSPCs (Zhao et al., 2015; Doro et al., 2017).

The specific embryonic origin of progenitor cells for suture is unknown. However, an integrated transcriptome and network analysis conducted by Holmes et al. (2020), and a single-cell resolution analysis performed by Farmer et al. (2021), identified *Lgr5, Lrig1*, *Prx1, Erg, Six2,* and *Pthlh*, as potential embryogenic Su-SSPCs markers. *Prx1* and *Shh* are also detectable in postnatal Su-SSPCs (Holmes et al., 2020; Farmer et al., 2021). The relationship between embryonic osteoprogenitor cells and postnatal Su-SSPCs, and the timing of the transition, remain to be explored (Holmes et al., 2020). Currently, four markers have been verified to label Su-SSPCs, namely,: 1) *zinc finger protein glioma-associated oncogene 1* (*Gli1* ^+^), 2) *axis inhibition protein 2* (*Axin2* ^+^), 3) *cathepsin k* (*Ctsk^+^*), and 4) *paired-related homeobox protein 1* (*Prx1* ^+^)-expressing cells (Zhao et al., 2015; Maruyama et al., 2016; Wilk et al., 2017; Debnath et al., 2018; Li et al., 2021). While it is not clear whether these four markers label the same Su-SSPC subset or they are mutually distinguishable, there has been a significant advance in the signaling pathways and potential interplay mechanisms in the regulation of Su-SSPCs.

A heterozygous loss of function mutation in *Twist1*, a basic helix–loop–helix transcription factor, results in reduced *Jagged1* expression and causes suture cells to become osteogenic (*Notch2* with *Runx2* expression) and original osteogenic cells to invade the suture (Yen et al., 2010; Ishii et al., 2015). In addition, this phenotype can be augmented by an accompanying specific *FGF* and *Gli3* mutations because a compound *Twist1*-*Gli3* mutation results in aberrant *Runx2* expression in sutural cells (Ishii et al., 2015). Interestingly, compound *Twist1-EphA4* heterozygotes show loss of the osteogenic-non osteogenic boundary integrity of the coronal suture, suggesting the role of *EphA4* in the migration of osteogenic cells to the leading edges of bone fronts (Ting et al., 2009; Ishii et al., 2015).

The *Fgfr* and *Gli3* signaling is known to maintain cell stemness during limb development. Consistently, a missense mutation in *Fgfr2* leads to suture mesenchyme ossification (Bonaventure, 2001; Ishii et al., 2015). Physiologically controlled by Hh signaling, *Gli3* acts like one end of a transcriptional switch with *Gli1* and *Gli2* transcription factors, and suppresses osteogenic differentiation. Without Hh signaling, non-mutated *Gli3* is active, inhibits transcription of certain genes (e.g., *Gli1, Gli2, Ptch1, Ccnd1, Igf2, Myc*, and *Bcl2*), and maintains cell stemness (McCubrey et al., 2014). *Gli1* ^+^ Su-SSPCs*,* therefore*,* contribute to calvarial bone formation through Hh signaling regulation. Treatment with IHh significantly upregulates *Gli1* ^+^ Su-SSPCs differentiation, whereas IHh signaling antagonist GDC0449 significantly downregulates *Gli1* ^+^ Su-SSPCs differentiation (Zhao et al., 2015). In injury experiments, *IHh* knock-out resulted in decreased bone volume and osteoporosis (Zhao et al., 2015). More recently, Greenblatt and others knocked out *Twist1* in *Ctsk* ^*+*^ lineage cells to create a craniosynostosis model. Unexpectedly, they observed that the cells expressing *Discoidin domain-containing receptor 2 (DDR2)* populate the suture with a corresponding decrease in *Ctsk* ^+^ Su-SSPCs, and proposed that these are a distinct population of Su-SSPCs (Greenblatt et al., 2021)*.*

The BMP and Wnt pathways are also fundamental to the development of calvarial bones and sutures (Maruyama et al., 2010; Ishii et al., 2015). In *Axin2* ^+^ Su-SSPCs, BMP signaling in presence of both *Axin2* and *type 1a BMP receptor* (*Bmpr1a*) expression suppresses early neonatal osteogenesis and maintains their stemness. *Rap1b*, a signaling effector of *Axin2*, mediates the balance between chondrogenic BMP to osteogenic FGF effect in the postnatal Wnt signaling pathway (Maruyama et al., 2010; Maruyama et al., 2017; Maruyama et al., 2021). Postnatal *Prx1* ^+^ (pn*Prx1* ^+^) Su-SSPCs also respond to Wnt signaling. Transcription factor profiling under physiologic conditions showed high levels of Wnt inhibitors, *Dkk1* and *Sost*, in pn*Prx1* ^+^ Su-SSPCs. Furthermore, inactivated Wnt signaling maintains the undifferentiated quiescent status of pn*Prx1* ^+^ Su-SSPCs, suggesting that Wnt signaling activation allows calvarial bone development and remodeling through pn*Prx1* ^+^ Su-SSPCs differentiation (Wilk et al., 2017). Given that pn*Prx1* ^+^ SSPCs are also found in the periosteum of long bones (Esposito et al., 2020), it is possible that pn*Prx1* ^+^ periosteal SSPCs are present and contribute to the observed long bone injury remodeling process as well.

Below the sutures, there is a fibrous membrane called the dura mater that provides paracrine signals for skull bone expansion and healing upon injury (Levi et al., 2011; Wang et al., 2020). The dura mater cells release BMP that serves as a stimuli to suture cells during brain and bone expansion (Levi et al., 2011) (Jin et al., 2016). After an injury, there is upregulation of *BMP2/4/7*, *FGF-2*, *FGFR-1*, *IGF-2* and *Ptn*, osteogenic markers *Sparc* and *Oc*, and osteoclast activity markers *Acp5*, *Ctsk, Mmp2,* and *Mmp14* in dura mater cells (Wan D. C. et al., 2008). Additionally, the absence of dura leads to fusion of the coronal suture, supporting the regulatory role and interaction of the dura with Su-SSPCs (Opperman et al., 2009). As sutures close, the reservoir of Su-SSPCs is lost. Given this closure or fusion of calvarial sutures happens physiologically during adulthood or prematurely with craniosynostosis, spontaneous repair of the critical-sized calvarial defect is a rare phenomenon in adults. To our knowledge, there is only one reported total re-ossification case in the adult age to date (González-Bonet, 2021).

In summary, the *Twist1* regulatory network, *EphA4* signaling*,* Hh/*Gli* pathway, and FGF receptors are uniquely involved in the development and patency of calvarial sutures. However, much still remains to be determined with regard to the regulatory mechanisms and their interactions, as well as the cellular processes in place. Su-SSPCs contribute to bone healing after injury; however, a slower rate is observed in the healing of defects in the calvarial periosteum compared to long bone periosteum (Lim et al., 2013). The absence of muscles and tendon attachments in the cranial region, which provides an additional layer of regulation, may be one of the reasons for this delay of healing.

### 3.2 Unique regulation of growth plate SSPCs (GP-SSPCs)

The GP consists of cartilaginous tissue that has a critical role in endochondral bone formation and elongation (Matsushita et al., 2020b). It is composed of three different layers with the resting zone on top (Ono and Kronenberg, 2016). It has long been thought that cells in the resting zone do not divide (Gibson, 1998; Shapiro et al., 2005), but recent studies demonstrated that, upon formation of the highly vascularized secondary ossification center, a postnatal SSPC niche is established in the resting zone located at the epiphysis of long bones (Mizuhashi et al., 2018; Newton et al., 2019).

Ono and others discovered postnatal chondrocyte cell populations expressing *PTHrP–mCherry* in the resting zone with SSPC markers ([CD45^−^Ter119^−^CD31^−^CD51^+^CD90^−^]CD105^−^CD200^+^ mouse SSCs, CD105^−^CD200^−^ pre-bone, cartilage and stromal progenitors [pre-BCSPs], and CD105^+^ BCSPs). These *PTHrP* ^+^ SSPCs give rise to transit-amplifying chondrocytes in the proliferating zone and to columnar chondrocytes from the early postnatal age decreasing until 6 months. The columnar chondrocytes undergo hypertrophy and subsequently differentiate into osteoblasts and *Cxcl12* ^+^ BMSCs beneath the GP (Mizuhashi et al., 2018). Similarly, Zhou and others observed migration of perinatal GP chondrocytes to the metaphysis just below the GP which form new osteoblasts in the BM and periosteum until 2 months of age, with significant decrease in contribution after adolescence (Shu et al., 2021). Resting *PTHrP* ^+^ SSPCs secrete PTHrP, which binds to the receptors expressed on the proliferating chondrocytes. This suppresses their hypertrophic differentiation and delays the production of IHh derived from pre-hypertrophic chondrocytes. As proliferation progresses, the distance between the resting and the hypertrophic zones increases, which naturally releases the PTHrP-induced IHh suppression. Despite the presence of IHh, BMP acts as a downstream regulator of proliferation; IHh and BMP act in a positive feedback loop, allowing an increased rate of chondrocyte proliferation and inhibition of the development of terminally differentiated chondrocytes. *Runx2* also positively regulates *IHh* expression and promotes chondrocyte proliferation (Mizuhashi et al., 2018; Ağırdil, 2020). Consistent with calvarial sutures, local FGF antagonizes BMP activity, which results in the downregulation of proliferation, and promotion of differentiation and columnar chondrocyte formation in the area (Mizuhashi et al., 2018; Ağırdil, 2020; Matsushita et al., 2020b).

Another GP-SSPC population, *Col2a1* ^+^, was identified using a multicolor fluorescent reporter and *Col2a1-CreER*. *Col2a1* ^+^ SSPCs were present but few at E14.5 and early postnatal age, only increasing markedly at P30. The mammalian target of rapamycin complex 1 (mTORC1) signaling shifts the cell division of resting zone *Col2a1* ^+^ SSPCs from asymmetric to symmetric, which results in increased number and thickness of multi-columnar clones. Activation of mTORC1 may be a response to changes in local energy and oxygen levels (Newton et al., 2019). Moreover, insulin-like growth factor 1 (IGF-1) signaling induces mTOR signaling activity and suppresses PTHrP production (Ağırdil, 2020). Since the GP is expected to be highly active during early human development until puberty, it is logical that increase of GP-SSPCs for bone formation would be triggered by mTOR-activating factors such as physical loading and muscle hypertrophy due to an active lifestyle and a protein-rich diet. *Axin2* ^+^ cells were also identified in the outermost layer of the GP acting as chondroprogenitors (Matsushita et al., 2020b). While in the resting zone, Wnt-inhibitory environment allows the maintenance of *PTHrP* ^+^ GP-SSPCs and activation promotes cell proliferation without columnar formation (Hallett et al., 2021), Wnt activation in the GP periphery promotes chondrocyte formation of *Axin2* ^+^ GP-SSPCs (Matsushita et al., 2020b). This possibly explains GP lateral expansion, but the physiological triggers of activation of Wnt/β-catenin remain to be investigated.

G-protein-coupled receptors (GPCRs) also regulate the self-renewal and differentiation capabilities of resting zone chondrocytes. Global ablation of Gsα (*Col2a1-creER; Gnas f/f*) causes premature differentiation of stem-like resting chondrocytes. When combined with the inactivation of Gq/G11α, a more severe phenotype with GP fusion occurs (Guo et al., 2009). These results suggest that PTH/PTHrP receptor-mediated protein stimulatory subunit-α (Gsα) and Gq/G11α synergistically maintain the quiescence of resting chondrocytes and their differentiation into columnar chondrocytes (Chagin et al., 2014). However, details of this regulatory mechanism, and whether this occurs without a trigger in resting chondrocytes, are yet to be determined.

Recently, periosteal cells were reported to interact with GP cells and regulate their endochondral bone formation. Periosteal osteoblast-derived *Osteocrin* (*OSTN*) inhibits *Natriuretic peptide receptor 3 (NPR3)* expressed in the GP hypertrophic chondrocytes. *OSTN* released by periosteal osteoblasts is delivered to the GP possibly through the epiphyseal and metaphyseal arteries supplying the ends of the GP. When active, *NPR3* causes the degradation of C-type natriuretic peptide (CNP) of the CNP-guanylate cyclase (GC)-B signaling pathway which is expressed in proliferating and pre-hypertrophic zones of the GP. Given that CNP promotes chondrocyte proliferation and maturation, the inhibition of *NPR3* by *OSTN* from periosteal osteoblasts produces a pro-chondrogenic effect (Potter et al., 2006; Watanabe-Takano et al., 2019). Periosteal osteoblast production of *OSTN* decreases with age, entirely downregulated by 3-month in mice (Watanabe-Takano et al., 2019). With GP chondrocytes forming BM and periosteal bone, positive feedback loop between the periosteal and GP cells seems to be in play contributing to bone elongation during early postnatal development.

In summary, long bone elongation *via* endochondral ossification is highly complex and structured, with chondrocytes taking a central role in the process. Being an analogous structure to calvarial sutures, similarities in regulations are evident. The GP eventually closes near the end of puberty (Setiawati and Rahardjo, 2019) and is regulated by the same pathways mentioned above. A decrease in the proliferative capacity of the SSPCs in the resting zone, together with decreased production of extracellular matrix (ECM), leads to GP closure and a limited contribution of trabecular osteoblasts in adult life (Ağırdil, 2020).

### 3.3 Unique regulation of periosteal SSPCs (P-SSPCs)

The periosteum, composed of the fibrous outer layer and cambium inner layer, covers the outer surface of the cortical bone. Periosteal SSPCs and osteoblasts are considered to be housed in the cambium layer (Matsushita et al., 2020c) and contribute to bone thickening and cortical maintenance during development and homeostasis (Serowoky et al., 2020). In addition, these periosteal cells are required for bone appositional growth, which occurs throughout life due to stress from increased muscle activity or weight.

Recently, *Ctsk* ^+^ cells with the SSPC immunophenotype (CD45^−^ TER119^−^ CD31^−^ THY^−^ 6C3^−^ CD200^+^ CD105^−^) were identified and located in the periosteum of postnatal long bones and calvaria (Chan et al., 2015; Debnath et al., 2018). However, the *Ctsk* gene transcribes cathepsin K, a thiol protease that is highly expressed in osteoclastic cells. Thus, it should be noted that *Ctsk* is not a specific P-SSPC marker and *Ctsk* ^+^ cells include tartrate-resistant acid phosphatase (TRAP)-positive osteoclastic cells in the BM and TRAP-negative SSPCs and osteoprogenitor cells in the periosteum (Debnath et al., 2018; Zhang et al., 2022). *Axin2-CreER* also labels a subpopulation of postnatal P-SSPCs (Maruyama et al., 2016; Ransom et al., 2016), but non-specifically marks the endosteal cell population as well (∼42%) (Ransom et al., 2016). Aside from Wnt signaling, *liver kinase b1* (*LKB1*), a master serine/threonine kinase and known tumor suppressor that links energy homeostasis and cell growth through the mTORC1 pathway, may also play a role in the maintenance of *Ctsk* ^+^ P-SSPCs. Studied in osteosarcoma formation, deletion of *LKB1* in *Ctsk* ^+^ P-SSPCs resulted in increased mTORC1 activity, subsequently causing an osteogenic tumor-like phenotype (Han et al., 2019). Therefore, the presence of *LKB1* may promote P-SSPC quiescence, while mTORC1 activation promotes appositional growth. The involvement of mTORC1 in both GP- and P-SSPCs suggests the importance of both axial and lateral bone growth during bone development.

In an attempt to define a specific marker for more immature osteogenic progenitor cells in adult bones, Park et al. defined *Mx1-Cre* as an efficient labeling model for osteogenic stem/progenitor cells (Park et al., 2012). In a subsequent study, they showed that double labeling of *Mx1-Cre* with *αSMA*GFP^+^ allows selective labeling of endogenous P-SSPCs (Ortinau et al., 2019). This P-SSPC population expresses CCR5, which results in their migration to injury sites with increased CCL5 from immune cells. Further, they showed that immune cells specifically from the macrophage lineage seem to play an important role in supporting periosteal niches. A deficiency in cytokine colony-stimulating factor 1 (CSF-1) in mononuclear cells, macrophages, and osteoclasts lead to a significant reduction of *Nestin*-, *Osx*-expressing, and *Leptin receptor (LepR)*-traced cells (Gao et al., 2019), which further supports the presence of interactions among cells within skeletal compartments.

*Mx1^+^αSMA* ^GFP^+^^ P-SSPCs are present in long bones and calvaria, and they overlap with pn*Prx1* ^+^ periosteal cells (Ortinau et al., 2019). Consistently, most *Prx1* ^+^ SSPCs are present in the long bone periosteum during embryonic and postnatal development. *Prx1* ^+^ cells are present during embryonic development restricted to the mesoderm which becomes mesenchymal cells postnatally without losing their embryonic tissue specification and thus have SSPC properties (Du et al., 2013; Bragdon et al., 2022). These pn*Prx1* ^+^ P-SSPCs are known to inhibit adipogenesis by activating TGFβ signaling (Du et al., 2013). Furthermore, pn*Prx1* expression and osteogenic activity of pn*Prx1* ^+^ P-SSPCs are induced in long bone injuries. Gene ontology study showed that they serve as a subset of reserve stem cells with the expression of stemness and limb development genes that can be engaged in tissue remodeling following injury (Duchamp de Lageneste et al., 2018). Injury-induced postnatal expression of *Prx1* in the periosteum is regulated by the BMP/CXCL12 interaction. Increases in BMP2 after injury result in a decrease in CXCL12 and *Prx1*, and *vice versa*. On day 14 post-injury, BMP2 upregulation leads to a decrease of CXCL12 expression and downregulation of *Prx1*, allowing cells to commit to callus maturation and osteogenic differentiation (Esposito et al., 2020).

The regenerative potential of P-SSPCs was also shown to be controlled by *Periostin (Postn)*. A microarray analysis of pn*Prx1* ^+^ P-SSPCs isolated from non-injured and injured bone identified that the *Postn* gene, expressed within the periosteum, is important for both intramembranous and endochondral re-ossification (Duchamp de Lageneste et al., 2018). *Postn* is a matricellular protein regulating cell-cell and cell-matrix interactions (Bonnet et al., 2009; Bonnet et al., 2013) and, when knocked out, causes reduced callus size, abnormal repair of unicortical bone defects that heal through direct bone formation, reduced bone volume throughout the repair, and local deficiency in the P-SSPC pool. *Postn* and its linked genes contribute to P-SSPC activation, niche regulation, and production of ECM proteins in response to bone injury (Duchamp de Lageneste et al., 2018). Similar to *Postn*, *Sclerostin domain-containing protein 1* (*Sostdc1*), a BMP and Wnt signaling antagonist primarily expressed in the periosteum, also maintains the P-SSPC pool (Semënov et al., 2005; Yanagita, 2005). The absence of *Sostdc1* hastens the expansion and differentiation P-SSPCs during bone healing (Collette et al., 2013).

Under normal homeostatic conditions, P-SSPCs provide a cellular source for the maintenance and growth of periosteal bones inherently through intramembranous ossification. However, these cells are able to undergo endochondral fracture repair with the formation of cartilage intermediates. *Mx1^+^αSMA* ^*GFP* ^+^^ P-SSPCs demonstrated this plasticity triggered by injury (Ortinau et al., 2019). Although the exact mechanism as to how this occurs is yet unknown, extracellular lipids, the hypoxia-inducible factor-1α (HIF-1α) and the BMP signaling pathways may be involved in this process (Hanada et al., 2001; Eyckmans et al., 2009; van Gastel et al., 2020; Zhang et al., 2022). The avascular state of the injury limits serum supply and creates extracellular lipid scarcity which activates FoxO signaling and pro-chondrogenic *Sox9* expression in the P-SSPCs (van Gastel et al., 2020). During bone repair, the HIF-1α pathway, required for normal skeletal development, is also activated (Wan C. et al., 2008) and is coupled with the action of VEGF, which is released by hypertrophic chondrocytes as well as osteoblast and undifferentiated cells near the injury (Wan C. et al., 2008; Wan et al., 2010; Nagao et al., 2017). This can initiate the invasion of blood vessels and facilitate GP- and P-SPPCs regulatory interactions. VEGF which is known to be a chondrocyte survival factor during development and bone formation (Nagao et al., 2017) could initially support the cartilage intermediate formation until enough vasculature and lipid levels are present for subsequent osteogenesis of remaining adjacent P-SSPCs.

In summary, postnatal P-SSPCs are heterogenous populations with unique regulatory mechanisms. Due to their proximity with the GP and BM niches, P-SSPCs may interact with cells from other compartments, thus affecting their regulation and contribution to osteochondrogenic bone regeneration. The identification of other regulatory factors or selective control mechanisms of P-SSPCs will present promising new approaches for bone regeneration.

### 3.4 Unique regulation of bone marrow SSPCs (BM-SSPCs)

The BM contains distinct SSPC populations with self-renewal and multi-lineage differentiation potentials (Herrmann and Jakob, 2019). BM-SSPCs are critical niche constituents with hematopoiesis-supportive function (Dominici et al., 2006), and are spatially associated with hematopoietic stem cells (HSCs) (Méndez-Ferrer et al., 2010). The BM is more prominent in long bones as compared to the calvarial bones, and the interaction between BM-SSPCs and HSCs is also more pronounced in long bones (Sadr et al., 1980; Chan et al., 2009; Chan et al., 2013; Ma et al., 2015).

As mentioned earlier, BM forms during bone development with blood vessels invading through a layer of committed osteogenic cells. *Osx* ^+^ and *Dlx5* ^+^ osteogenic precursor cells populate the forming fetal marrow with the development of the blood vessels (Maes et al., 2010; Liu et al., 2013; Matsushita et al., 2022). While both cells contribute to fetal periosteum and marrow stroma development, *Osx* ^+^ cells are transient as their number dramatically declined after 13 weeks, leaving *Dlx5* ^+^ cells as the major BM-SSPCs with the role of regulating BM space formation (Mizoguchi et al., 2014; Matsushita et al., 2022). *Fgfr3* ^+^ cells contributing to the fetal cartilage template and fetal GP also form BM-SSPCs in embryonic trabecular bone formation, together with a subset of *Osx* ^+^ and *Dlx5* ^+^ cells. Mechanistically, IHh secreted by *Fgfr3* ^+^ cells bind to the *Ptch1* of *Dlx5* ^+^ BM-SSPCs to promote BM space formation. Similar to its effect on GP chondrocytes, secreted IHh also promotes proliferation of *Fgfr3* ^+^ BM chondrocytes which may differentiate into osteoblasts (Matsushita et al., 2022).

Postnatally, *Dlx5* ^+^ cells localize in the mid-diaphysis retaining its BM-SSPC properties but with adipogenic tendencies to become *Perilipin* ^+^ marrow adipocytes in adult bones. Interestingly, a subset of *Fgfr3* ^+^ cells develop into postnatal metaphyseal BM-SSPCs with osteogenic tendencies contributing to *alkaline phosphatase*-expressing osteoblasts (Shu et al., 2021; Matsushita et al., 2022). These cells may be the same population as the *PTHrP* ^+^ GP hypertrophic chondrocytes that turns into *Cxcl12* ^+^ BM-SSPCs beyond the GP (Mizuhashi et al., 2018). Separately, postnatal *LepR* ^+^ BM-SSPCs with *Osx* expression are responsible for new osteoblasts in adult BM and in the metaphyseal area (Mizoguchi et al., 2014; Shu et al., 2021). While a portion of the this cell population differentiate to trabecular osteoblasts, some cells remain unchanged in the metaphyseal stroma with long term SSPCs properties, and a portion change into BM reticular cells (Maes et al., 2010; Liu et al., 2013; Mizoguchi et al., 2014; Matsushita et al., 2020c; Matsushita et al., 2020a). These BM-SSPCs proliferate along the developing blood vessels regulated by the endothelial cell-derived PDGF-BB signaling pathway through PDGFRβ of the precursor cells, which subsequently become perivascular cells that establish the BM stroma (Bianco et al., 2013). These cells are marked as CD45^−^/CD34^−^/CD146^+^, with the *Osx* expression confirming its osteogenic origin (Liu et al., 2013). Consistently, a perivascular cell marker *Nestin-GFP* also labels BM-SSPCs with stem cell functions at E15.5 and postnatal to adulthood, supporting the idea that at least a subset of BM-SSPCs has BM perivascular location (Méndez-Ferrer et al., 2010; Wei and Frenette, 2018).

*Gli1* ^+^ metaphyseal mesenchymal progenitors (MMPs) located beneath the GP express SSPC markers CD146, CD44, CD106, and CD140a (PDGFRα), and may possibly label the same population of unchanged postnatal *Osx* ^+^ osteogenic precursor cells in the metaphyseal region. Both cells migrate from the perichondrium to the BM at E15.5–16.5, suggesting cell population overlap even during embryonic development (Shi et al., 2017; Matsushita et al., 2022). Proliferation and osteoblast differentiation of *Gli1* ^+^ MMPs is driven by β-catenin and *Hh* signaling from the pre-hypertrophic chondrocytes of the GP. Without β-catenin (e.g., GP closure), adipogenesis is favored and *LepR* expression is observed. These early *Gli1* ^+^ progenitor cells also disappear from their position in aged mice and do not contribute to major *Cxcl12* ^+^ stromal cells (Shi et al., 2017), implicating that they are more likely osteochondrogenic progenitor cells rather than SSCs. Populations of SSPCs that express *Gremlin1* (*Grem1*), a secreted BMP antagonist, were also identified in the embryonic and postnatal mice (Chan et al., 2015; Worthley et al., 2015). Postnatal *Grem1* ^+^ cells in the BM metaphysis, just under the GP, define a population of osteochondroreticular (OCR) stem cells with self-renewal, and osteoblasts, chondrocytes, and reticular BM-SSPC differentiation capacity during early development. Interestingly, these OCR stem cells do not differentiate into adipocytes. Deletion of *Grem1* results in BM hypoplasia with early hematopoietic failure (Rowan et al., 2020). From its properties and location, overlap with *Fgfr3* ^+^ BM-SSPCs is possible, but is yet to be established.

#### 3.4.1 Unique adipogenic regulation of BM-SSPCs

In the postnatal and adult mouse BM, most perivascular BM-SSPCs acquire marker expression such as *LepR* and *Mx1*, with the latter labelling not only stromal cells but HSCs as well (Park et al., 2012; Zhou et al., 2014). In addition, BM-SSPCs distinctly express cytokines responsible for the retention of hematopoietic progenitors such as CXCL12 or stromal cell-derived factor 1 (SDF1), stem cell factor (SCF) (Wei and Frenette, 2018). *LepR* ^+^ cells are observed only in postnatal perisinusoidal or periarteriolar BM-SSPCs (Zhou et al., 2014; Shen et al., 2021) that are largely overlapping with the *Cxcl12*-abundant reticular (CAR) cells (Matsushita et al., 2020a). In addition, CAR cells have subclusters and have been reclassified into osteo-CAR (*Cxcl12* ^*+*^ *Osx* ^*+*^) and adipo-CAR cells (*Cxcl12* ^*+*^ *LepR* ^+^), having pre-osteogenic and pre-adipogenic tendencies respectively (Matsushita et al., 2020a; Baccin et al., 2020; Shen et al., 2021). Recently, a mechanosensitive *LepR* ^+^ *Osteolectin* ^+^ (*Oln* ^+^) cell population, a potential subset of the osteo-CAR population, has also been discovered, and are distinguishable from adipo-CAR population. They contribute to bone formation during injury and mechanical loading through the mechanosensitive ion channel, Piezo1 (Shen et al., 2021). The *Fgfr3* ^+^ metaphyseal and *Dlx5* ^+^ diaphyseal BM-SSPCs were also proposed to represent osteo- and adipo-CAR cells, respectively, implying that these two CAR cell populations developed from two distinct origin sharing the same marker rather than coming from a single progenitor (Matsushita et al., 2022). Further, these osteo- and adipo-CAR cells have a distinct periarteriolar and perisinusoidal location respectively, implicating their heterogeneity. Osteo-CAR cells contributes to cortical bone formation during homeostasis and injury regeneration, while adipo-CAR only minorly contributes during injury repair (Matsushita et al., 2020a; Baccin et al., 2020; Shen et al., 2021). During homeostasis, adipo-CAR cells express potent Wnt inhibitors such as *Sfrp1*, *Sfrp2*, and *Sfrp4,* suggesting a role for *Cxcl12* in the inhibition of CAR cell differentiation (Matsushita et al., 2020a). *Foxc1* and *Early B-cell factor1/3* (*Ebf1/Ebf3*) also contribute to this inhibition and are important in the maintenance of BM-SSPCs. Deficiency of *Foxc1* or *Ebf1/3* in *LepR* ^*+*^ cells results in osteosclerotic BM, impaired HSC niche function, and fibrotic conversion of the BM-SSPCs (Seike et al., 2018; Omatsu et al., 2022). Upon injury, activation of Wnt signaling may stimulate production of BMP, which interacts with type Ib receptor Bmpr (Bmpr1b) (Muruganandan et al., 2009). Additionally, pre-adipogenic factors are inhibited, which may further stimulate their osteogenic differentiation (Abdallah, 2017). However, whether this plasticity is due to a bipotential capacity or presence of a quiescent osteogenic progenitor subset is still unknown.

Reconciling rare adipocytes in the young postnatal marrow, Zhong et al. (2020) reported a group of non-proliferative cells expressing adipocyte genes (e.g., *adiponectin* [*Adipoq*]) called marrow adipogenic lineage precursor (MALP) cells. These MALPs lack significant lipid stores usually seen in adult adipocytes and lack adipocyte progenitor markers such as *SCA1* and *CD34*. Interestingly, MALPs form a vast 3D network structure inside the BM that allows cell-to-cell contact and BM environment interaction which may be important for marrow vasculature maintenance and suppression of osteogenic differentiation. Early adiponectin studies reported that adiponectin can facilitate the migration of osteoblast progenitors to the endosteal injury site through increasing sphingosine1 phosphate (S1P) (Li et al., 2009; Holland et al., 2011) since osteoblasts are reported to express the S1P receptor (Sartawi et al., 2017). Conversely, adiponectin repels osteoclast progenitors and osteoclasts from injury sites, allowing structured intramembranous bone repair (Yang et al., 2015). However, recent studies showed that the removal of *Adipoq-Cre* ^*+*^ cells resulted in disruption of sinusoidal vessels and a significant increase in bone trabeculae in the marrow space (Zhong et al., 2020). Further, *Adipoq-Cre* ^*+*^ MALPs highly express *Cxcl12*, *Scf*, and Csf1 (Zhong et al., 2020; Yu et al., 2021) needed for HSC retention, hematopoietic regeneration after injury, and osteoclast activation, respectively (Peled et al., 1999; Zhou et al., 2017; Yu et al., 2021). These factors attract osteoblast and osteoclast progenitors expressing CXCR4 into the BM, supporting a role of *Adipoq-Cre* ^*+*^ cells in the bone marrow function. Whether MALPs and adipo-CAR cells overlap or are distinct cell populations is still unknown. Further elucidation of the role of adipo-CAR cells during homeostasis may therefore reveal novel functions in BM maintenance and osteoblast regulation (Ortinau and Park, 2021).

#### 3.4.2 Unique hematopoietic regulation of BM-SSPCs

The BM is an essential environment for HSPCs. In particular, BM-HSCs are in a fluid condition and require the niche interaction with perivascular SSPCs through adhesion proteins (e.g., *Scdf1* and E-selectin) for their long-term maintenance (Sipkins et al., 2005). Conversely, HSPCs contribute to the maintenance, as well as the activation, of their niche cells and BM-SSPCs *via* their inflammatory cells. Th1 cells secrete TNFα, which mediates increased RANKL expression by macrophages and B-lymphocytes (Lam et al., 2000; Castillo et al., 2017). These increased RANKL expression from B-lymphocytes can control osteoclastogenesis (Walsh and Choi, 2014; Toni et al., 2020). Further, B-lymphocytes have spontaneous production of granulocyte-colony stimulating factor (G-CSF) throughout life and increased Osteoprotegerin (OPG) production with age (Li et al., 2015) with boosts under the stress or inflammatory conditions (Corcione and Pistoia, 1997). Depending on the expression levels of G-CSF and OPG, osteogenesis (high G-CSF, low OPG) or osteoclastogenesis (low G-CSF, high OPG) may be favored (Weitzmann, 2013; Zhang et al., 2020). Therefore, B-lymphocytes are an important regulator of SSPCs by contribute to bone healing after injury and excessive bone resorption during aging.

M1 macrophages also contribute to SSPC maintenance and activation. They secrete reactive oxygen species (ROS), nitric oxide (NO), and several proinflammatory cytokines (e.g., IL-1, IL-2, IL-6, TNFα, and IFNγ) (Genin et al., 2015). NO allows vasodilation which may increase the migration of cells through and from the BM (Bianco, 2011). Together with factors such as IL-1 and IL-6, which decrease osteogenic differentiation, these macrophages further help in the establishment of the BM niche and maintenance of BM-SSPCs. On the other hand, M2 macrophages secrete pro-osteogenic molecules BMP2, TGFβ, and osteopontin (Chen et al., 2020). Additionally, activated neutrophils produce IL-1α and TGFβ directly causing BM-SSPC differentiation into osteoblasts (Al-Hakami et al., 2020) and inhibition of ECM production (Bastian et al., 2018).

Overall, the interaction of HSPCs and BM-SSPCs does not only affect the BM niche but bone turnover as well. While the identity of BM-SSPCs remains elusive, the regulation mechanisms of BM-SSPCs appear to be highly connected to factors released from the GP chondrocyte and BM inflammatory cells, suggesting that cellular and molecular regulators interact across skeletal compartments (Figure 4).

## 4 Regulations during aging

The elderly population has poor capacity for skeletal regeneration and a limited physiologic SSPC reserve (Lee et al., 2014) leading to degenerative conditions (Jeong and Park, 2020). SSPCs from older individuals have similar clonogenicity, but impaired osteochondrogenic differentiation, as compared to younger individuals (Ambrosi et al., 2020). Changes in hormones and sustained pro-inflammatory stimuli in aging might alter epigenetics regulators (Beerman and Rossi, 2015; Josephson et al., 2019). A recent study showing the downregulation of histone deacetylase *Sirtuin1* in aged human SSPCs supports this hypothesis (Ambrosi et al., 2020). Moreover, downregulation of osteogenic genes (e.g., Wnt signaling), and upregulation of fibroblast-like ECM- and cellular senescence-related genes, were seen in aged human SSPCs, suggesting skewing towards stromal/fibroblastic states (Ambrosi et al., 2020). Excess or continuous inflammation in the elderly, and low-grade chronic inflammation associated with degenerative and cardiometabolic diseases, are known to inhibit the regeneration of various tissues including bones (Franceschi et al., 2018; Josephson et al., 2019). Activation of Nuclear factor-kappa B (NF-κB), a regulator of innate immunity, resulted in increased expression of the senescence genes *Cdkn1a* and *Cdkn2a*, suggesting its central role as a mediator of a pro-inflammatory state and SSPC aging (Albensi, 2019) (Josephson et al., 2019). Extensive proliferation may also lead to cellular exhaustion. Sustained FGF2 signaling in SSPCs and increased neutrophil-related ROS in HSC cause loss of quiescence and impaired regenerative capacity of SSPCs (Owusu-Ansah and Banerjee, 2009; Chakkalakal et al., 2012).

With age, the rest of the BM undergoes adipocyte conversion where fat cells progressively increase in number. Adipocytes then inhibit BM-SSPC functions (Bianco, 2011). Normally, adipogenesis involves sequential expression of *CCAAT enhancer-binding protein beta* (*C/EBPβ*), *gamma* (*C/EBPγ*), *alfa* (*C/EBPα*), and finally *peroxisome proliferator-activated receptor gamma* (*PPARγ*) from progenitor cells. Several transcription factors direct age-related shifts in BM-SSPC differentiation. MAF bZIP transcription factor (MAF), a binding partner of *Runx2*, is increased in the young but decreased in the old. With age, reduced MAF promotes adipogenesis through upregulation of *PPARγ* and the suppression of osteogenesis (Nishikawa et al., 2010). *Forkhead box P1* (*FOXP1*) also declines with age, losing its anti-adipocyte interaction with *C/EBPβ* and pro-osteogenic repression of Notch signaling pathway, all leading to bone loss (Li et al., 2017). Core-binding factor subunit beta (CBFβ) is another key co-factor of *Runx2* that is reduced with aging. Normally, CBFβ inhibits adipogenic gene expression and enhances Wnt/β-catenin signaling (Wu et al., 2014; Wu et al., 2017). An increase of BM adipocytes with age is also associated with the gradual decrease of *Adipoq* ^+^ expression, potentially facilitating MALPs differentiation to adipocyte cells. Expression of *Cxcl12* is also reduced, further leading to BM atrophy and adipogenesis (Zhang et al., 2021). Furthermore, BM adipocytes may also inhibit BM-SSPCs function by physically blocking blood flow through the sinusoid. The larger-sized adipocytes can compress the sinusoid, leading to its collapse (Bachman et al., 2002).

Unlike most bones, cranial bones are rarely affected by osteoporosis. However, the same mechanisms leading to this condition can also reduce cranial bone mass density and regeneration capacity (Cotofana et al., 2018; Hudieb et al., 2021). Radiographic and histologic studies showed a decreased computed volume of the calvaria and a lateral expansion of the skull, favoring a skeletonized facial appearance in elderly individuals (Cotofana et al., 2018). Increased soft tissue laxity and decreased fat (Cotofana et al., 2016) can contribute to increased bone resorption in elder individuals due to decreased mechanical loading. The tensile strength of the dura also decreases with age; alterations in collagen fiber organization may cause this change in dura properties, which ultimately affects the ECM of the tissue (Zwirner et al., 2019; Panteleichuk et al., 2021). The osteogenic activity of the dura also tends to be less active with age (Wan D. C. et al., 2008), probably due to the absence of skull growth-induced mechanical strain (Wang et al., 2020).

Further understanding of which cellular and molecular changes SSPCs undergo during stress, aging, and pro-inflammatory conditions, and which regulatory mechanisms control these changes, will offer new approaches to the treatment of bone diseases through the ages.

## 5 Conclusion and future directions

Calvarial and long bones are unique types of bone that are distinctively regulated but show subtle similarities in the involved pathways. In both types of bone, multiple types of distinct SSPCs are present and interact with each other to achieve skeletal development. Despite improvements in our understanding of SSPCs, the different functional responses and regulations of SSPCs in various locations, especially during injury, have not been thoroughly studied. Although some essential molecular regulators are shared by distinct SSPCs, their effect on differentiation, cell fate, or tissue type formation of distict SSPCs can be different. As such, local SSPCs contribute uniquely to their bone development, homeostasis, and regeneration. Different conditions (e.g., injury, stress, aging) result in different regulations as well. Studies looking into these differences are currently inadequate. Possible regulatory differences directing the rate of defect or injury healing in the craniofacial area and long bones have not been extensively investigated despite this long-observed difference.

Long bone is important for addressing the mechanical loading throughout life, while craniofacial deformities are important not only for the physiology of the organs in the craniofacial area but also for the quality of life of patients in general. Thus, studies pertinent to enhancing healing of both long bones and craniofacial bones may have to be given equal importance. The unique effect of the limited presence of ligaments, tendons, and muscles in the craniofacial area, as compared to long bones, is an interesting area of research. Characterization of the heterogenous SSPC population in the BM, and the regulatory mechanisms by which they contribute to BM maintenance, could be continued and expanded as a research initiative. Single-cell approaches together with *in vivo,* and *ex vivo* functional studies appear to be a powerful approach to facilitate SSPC characterization and biology. Additionally, the single-cell approach would allow further analyses on differential gene expression and the regulatory mechanisms established between cell populations, skeletal compartments, and cell conditions (development vs. injury vs. aging).

Advancements in SSPC research and interest in the aging bone have allowed the discovery of more unique populations such as those adipocyte marker-expressing cells that do not undergo adipogenesis, but rather unexpectedly remains undifferentiated in the marrow with marrow stroma and cortical bone maintenance roles. Further research could also be done to deepen our understanding of how each multiple types of SSPCs relate to each in the context of development, regeneration, and aging. These differences are necessary for designing specific tissue engineering and regenerative medicine therapies for bone repair.
